# Editorial: Association of diabetes mellitus with cognitive impairment and neurological disorders, volume II

**DOI:** 10.3389/fendo.2026.1900873

**Published:** 2026-06-18

**Authors:** Kexin Zhang, Lei Sha, Xiaodong Sun

**Affiliations:** 1 Department of Endocrinology and Metabolism, Shandong Provincial Key Medical and Health Laboratory of Endocrinology and Metabolic Diseases, Affiliated Hospital of Shandong Second Medical University, Weifang, China; 2 Department of Neuroendocrine Pharmacology, School of Pharmacy, China Medical University, Shenyang, China

**Keywords:** Alzheimer’s disease, cognitive decline, cognitive impairment, diabetes, endocrine risk factors

Diabetes mellitus is a chronic metabolic disorder with hyperglycemia, insulin resistance, and vascular and inflammatory dysfunction, leading to vascular complications and cognitive decline, including Alzheimer’s disease and vascular dementia (1, 2). Cognitive deficits in diabetes often affect memory, executive functions, and attention, and are intertwined with cerebrovascular pathology, metabolic disturbances, and lifestyle factors (3–5). This Research Topic compiles 18 studies, including original research, reviews, and case reports, providing a comprehensive synthesis of epidemiology, molecular mechanisms, predictive modeling, pharmacological and lifestyle interventions, and methodological considerations in diabetes-related cognitive and neurological disorders. Collectively, these studies underscore the multifactorial nature of diabetic cognitive impairment and highlight opportunities for early identification, targeted intervention, and precision management.

Population-based and clinical studies consistently show that metabolic dysfunction predisposes individuals to cognitive deficits. Ge et al. reported a U-shaped association between BMI and cognitive impairment in middle-aged and older adults with type 2 diabetes mellitus (T2DM), with lowest risk at 23.72–27.77 kg/m² and elevated risk at both extremes, exacerbated by social isolation and reduced physical activity. Jiang et al. found that higher metabolic score for insulin resistance (METS−IR) correlated with memory and executive deficits, highlighting insulin resistance as a key determinant of cognitive vulnerability. Chen et al. reported that lower estimated glucose disposal rate, an index of insulin sensitivity, strongly predicted cognitive deficits in elderly T2DM patients. Wang et al. analyzed NHANES data, showing diabetes-associated cognitive deficits across racial groups, while Xu et al. confirmed diabetes as an independent risk factor for cognitive impairment in maintenance hemodialysis patients, developing a simplified predictive model (AUC 0.737). Wang and Sun further constructed a nomogram integrating age, diabetes duration, hyperglycemia, hypertriglyceridemia, and homeostasis model assessment of insulin resistance (HOMA-IR), achieving robust discrimination (C-index 0.868/0.710). These findings highlight that insulin resistance and chronic hyperglycemia are consistent determinants of cognitive decline across age groups and clinical contexts.

Metabolic derangements induce cognitive impairment through intertwined neuroinflammatory, vascular, and oxidative pathways. Yang et al. demonstrated that gut microbiota dysbiosis and metabolites exacerbate diabetic neuropathy and cognitive deficits in db/db mice, mediated by Dl-lactate and polygalic acid and involving NLRP3 inflammasome activation in dorsal root ganglia and hippocampal tissues. Huang et al. showed that elevated homocysteine increases cerebral small vessel disease burden via endothelial dysfunction, blood–brain barrier disruption, and oxidative stress, with synergistic effects from hyperglycemia and advanced glycation end-products confirmed experimentally. Wang et al. used Olink proteomics to distinguish Dys-NC (dysglycemia with normal cognition) from Dys-VCI (dysglycemia with vascular cognitive impairment), identifying pro-inflammatory proteins (CXCL1, TNFB, IL-12B) upregulated in Dys-VCI and anti-inflammatory proteins (FGF-21, AXIN1) downregulated. Kan et al. reviewed the epidemiology, mechanisms, and management of cognitive impairment in T2DM, emphasizing roles of insulin resistance, chronic inflammation, vascular injury, and oxidative stress. Chen et al. similarly highlighted hyperglycemia, neuroinflammation, oxidative stress, vascular injury, and gut microbiota dysbiosis as key contributors, noting molecular targets such as insulin-degrading enzyme, NLRP3, and ferroptosis pathways for potential neuroprotection. Collectively, these studies delineate the multifactorial mechanisms linking metabolic, vascular, and inflammatory dysregulation to cognitive decline in diabetes.

Pharmacological strategies demonstrate potential to mitigate cognitive decline beyond glycemic control. Lu et al. reported that DL-3-n-butylphthalide improved post-stroke cognition in non-diabetic patients, whereas benefits in diabetic patients were limited to modest improvements in orientation, indicating that metabolic status partially modulates therapeutic response. Song et al. systematically compared sodium–glucose cotransporter 2 inhibitors (SGLT2i) with incretin-based therapies, reporting lower dementia incidence with SGLT2i relative to dipeptidyl peptidase-4 (DPP−4) inhibitors, and comparable or slightly greater effects versus glucagon-like peptide-1 (GLP-1) receptor agonists. Giofrè et al. summarized that GLP-1 and glucose-dependent insulinotropic polypeptide (GIP) receptor agonists, as well as DPP-4 inhibitors, confer neuroprotection independently of glycemic control through anti-inflammatory, antioxidant, and synaptic plasticity-enhancing mechanisms, with dual GLP-1/GIP receptor agonists (tirzepatide) providing additional benefit via the gut–brain axis. Together, these studies highlight the therapeutic potential of metabolic-targeted pharmacologic interventions for preserving cognitive function in diabetic populations.

Clinical and mechanistic observations further illustrate the reversibility and context-dependence of metabolic brain dysfunction. Liu et al. described two elderly T2DM patients with acute diabetic striatopathy presenting choreiform movements and basal ganglia T1 hyperintensity, which fully resolved following glycemic control. Li et al. demonstrated that reduced bone mineral density and osteocalcin levels were independently associated with cognitive impairment, supporting the bone–brain axis as a mediator of metabolic-cognitive interactions. Wais et al. reported that children and adolescents with T1D exhibited impaired sustained attention, slower reaction times, and increased hyperactivity, particularly in patients with HbA1c >8%, highlighting the importance of early glycemic control during neurodevelopment. These findings emphasize that age, developmental stage, and metabolic control influence susceptibility and reversibility of cognitive deficits.

Rigorous methodological practices are essential for reliable inference in T2DM with mild cognitive impairment research. Liu et al. systematically reviewed observational studies and found that only 22.7% quantified missing data (mean 9.1%), 93.3% relied on complete-case analysis, and sensitivity analyses or mechanistic explanations were rare (4.4%), underscoring the potential for bias and the need for transparent reporting and standardized data handling.

Collectively, these studies provide a comprehensive view of T2DM-related cognitive impairment, spanning epidemiology, molecular mechanisms, pharmacological interventions, special populations, and methodological considerations. They emphasize early risk identification, targeted metabolic and lifestyle interventions, and attention to sociodemographic context. Future research integrating multimodal neuroimaging, longitudinal biomarkers, diverse populations, and precision therapeutics will be critical to advance prevention, monitoring, and individualized management strategies for cognitive and neurological complications in diabetes.
